# The impact of education through nurse-led telephone follow-up (telenursing) on the quality of life of COVID-19 patients

**DOI:** 10.1186/s42506-021-00093-y

**Published:** 2021-11-08

**Authors:** Rasoul Raesi, Zahra Abbasi Shaye, Sam Saghari, Mohammad Ali Sheikh Beig Goharrizi, Mehdi Raei, Kiavash Hushmandi

**Affiliations:** 1 Bahman Khaf Hospital, Mashhad University of Medical Sciences, Mashhad, Iran; 2 Department of Health Services Management, Tehran Medical Sciences, Islamic Azad University, Tehran, Iran; 3 Community Medicine, Akbar Clinical Research & Development Unit, Mashhad University of Medical Sciences, Mashhad, Iran; 4 Atherosclerosis Research Center, Baqiyatallah University of Medical Sciences, Tehran, Iran; 5 Health Research Center, Life Style Institute, Baqiyatallah University of Medical Sciences, Tehran, Iran; 6 Department of Food Hygiene and Quality Control, Division of Epidemiology & Zoonoses, Faculty of Veterinary Medicine, University of Tehran, Tehran, Iran

**Keywords:** Education, Telenursing, Quality of life, COVID-19

## Abstract

**Background:**

The widespread prevalence of COVID-19 has disrupted the social life, physical function, and daily activities of patients, leading to reduced quality of their lives. Because of the nature of this disease and its comprehensive impact on patients’ lives, a follow-up based on the conditions of these patients is necessary. This study was conducted to determine the impact of nurse education and telephone follow-up (telenursing) on the quality of life of COVID-19 patients.

**Methods:**

This quasi-experimental study included 120 COVID-19 patients discharged from 22nd-Bahman Hospital in Khaf city and was conducted over 6 months from July 20, 2020, to December 20, 2020. The participants were selected by convenience sampling method and were assigned into two matching groups. The training was delivered through telenursing based on the quality of life criteria for 1 month in the intervention group. The controls did not receive any intervention. Both groups completed the 36-item SF health survey before and 1 month after the intervention.

**Results:**

The two groups were not significantly different regarding the quality of life mean scores at baseline (*p* = 0.61). However, after the intervention, the mean and standard deviation of the total life quality score was significantly different between the control and intervention groups (63.62 ± 3.93 versus 72.62 ± 3.51, *p* <0.001).

**Conclusions:**

Telenursing improves the life quality of COVID-19 patients. Through appropriate policies, health managers may put on the agenda the implementation of telenursing for COVID-19 patients.

## Introduction

Coronaviruses are a large group of viruses belonging to the family of Coronaviridae, which range from the common cold virus to the pathogens of more serious diseases such as SARS, MERS, and COVID-19. The virus entered Iran in early March 2020 and has affected all provinces and cities. On March 11, 2019, the World Health Organization declared the disease a pandemic [1].

Patients with COVID-19 experience physical and mental distress, fear of complications, and discrimination [2, 3]. Besides, because COVID-19 patients are treated in separate hospitals, these negative feelings are intensified and can ultimately affect these patients’ quality of life (QoL) [4, 5]. The sudden onset of a disease has profound effects on mental health. The larger the scale of the disease, the greater the effects. Epidemics cause fear, and this fear can continue even after the disease is cured. Inadequate control over COVID-19 leads to hospitalization, high prevalence of the disease, and poor QoL of patients. The coronavirus and its complications cause economic burden, a decline in psychological QoL, and disruption in socio-familial relationships of the patient [6].

QoL is a complex concept that denotes people’s mental assessment of their well-being and ability to perform physical, psychological, and social functions. In other words, QoL can be defined by people’s perceptions of their position in life, value systems, and related standards, and is considered a component of health by researchers [7].

Health-related QoL is associated with fewer pathological symptoms, further societal behavior, and lower need for endorsement activities and can be influenced by demographic and social variables, diseases, and clinical status [8]. When a patient is linked with the healthcare system, s/he can receive the correct and continuous care needed, enjoy a shortened length of hospital stay, and have improved QoL [9, 10]. Conversely, if the healthcare system does not support the patient, it may cause many complications and exacerbate the disease [11].

As one of the main concepts of nursing, patient education is a health education approach to planning, providing, and evaluating healthcare, the purpose of which is to maintain the patient's integrity and provide unique care for each patient [12]. Therefore, education is indeed the fundamental contribution of nurses. Additionally, today’s nursing knowledge aims for and emphasizes self-care, empowerment, and enhanced QoL of patients and families. Therefore, education to patients and their families seems to be the best therapeutic approach [13].

There are several ways to educate patients and families. Traditional methods may not be fully responsive to changes and the rapid growth of information and educational needs of the community of patients with chronic diseases [14]. As an essential part of healthcare services to patients and families, regular remote follow-up or telenursing contributes to active involvement in the treatment process [15].

According to the International Association of Nurses, telenursing uses telecommunications technology in nursing to increase patient care, which includes the use of electromagnetic devices to transmit sound and information [10]. Telenursing is a subset of telemedicine [16]. Post-discharge telephone follow-up is a very useful and inexpensive method for assessing patients’ needs and helping callers’ care problems [17]. In addition, post-discharge telephone calls help identify and correct care gaps that may take place after discharge from the hospital [18].

Unfortunately, nursing education is assumed to be less significant by patients than other clinical practices, and educational interventions are often seen as unplanned and a major challenge for healthcare personnel [19]. However, studies on the use of telenursing as a telemedicine technology have revealed its positive contribution to QoL of patients with epilepsy in Malaysia [20], reduced risk of worsened diabetes in pre-diabetic individuals in Hong Kong [9], and improved self-efficacy in patients with rheumatoid arthritis in Denmark [21].

However, given the nature of COVID-19 as a pandemic with many potentially unknown dimensions, the disease can affect the life of the patient and his/her family in ways that are not yet known. Therefore, the question remains as to the effectiveness of telenursing on QoL of COVID-19 patients. This study was performed to determine the effect of education through telenursing on the QoL of COVID-19 patients.

## Methods

This quasi-experimental study used a pretest-posttest design and two groups. The study was conducted over 6 months, from July 20, 2020, to December 20, 2020. The study population comprised all patients with COVID-19 discharged from 22nd-Bahman Hospital of Khaf during the study period. Given the unavailability of a similar study, the sample size was computed as *n* = 120 upon the consensus of experts concerning the mean effect size of *p* = 0.5, alpha = 0.05, and beta = 0.2. Sampling in this study was continuous and was built on the convenience sampling method. The participants were assigned into intervention and control groups (*n* = 60 per group). Inclusion criteria were definitive diagnosis of COVID-19 with positive PCR, appropriate mental health as reported in psychiatric counseling at the time of admission, full consciousness, lack of speech and language problems, ability to self-care, and access to a phone at home. Exclusion criteria were reluctance to continue participating in the research and telephone calls unanswered three times. Explanations were given to the patients concerning confidentiality, voluntary nature of participation, and research objectives, procedure, and duration. They also announced their consent for participation.

The data collection instruments used in this study consisted of a demographics form (age, gender, marital status, occupation, and education) and the SF health survey (Ware and Sherborne, 1992) [22]. The survey holds 36 items and evaluates the eight domains of physical functioning, social functioning, role limitations due to physical problems, role limitations due to emotional problems, general mental health, vitality, bodily pain, and general health perceptions. A respondent’s score would range from 0 to 100 in each domain, and a higher score denotes greater QoL. The validity and reliability of this questionnaire have been confirmed in the Iranian population [23, 24]. The internal consistency coefficients of its domains range between 0.70 and 0.85, and the survey’s 1-week retest coefficients have been between 0.43 and 0.79. The survey can differentiate between healthy and unhealthy individuals in all domains [24].

Because this survey has not been used to assess the effectiveness of telenursing on QoL of COVID-19 patients, it was given to 10 nursing faculty members to confirm its validity, and the required modifications were applied in accordance with their opinions. Therefore, the content and face validities of the questionnaire were assured using expert opinion. To determine the internal consistency of this questionnaire, we computed the Cronbach’s alpha coefficient (*α* = 0.86).

The data collection instruments were completed for both groups over the phone before the educational intervention was initiated. The educational content was presented to the intervention group by phone based on the domains covered in the SF health survey [22]. The general content of the training program included the nature of COVID-19 disease, the type and amount of physical activity/exercise allowed, proper diet, disease aggravating factors and ways to control them, drugs (drug name, time of use, dosage, side effects, manner of use, etc.), re-visit (treatment follow-up), stress and anxiety control, and the right time to return to work.

Phone calls were made three times a week between 9 a.m. and 12 a.m., and the duration of each call ranged between 10 and 15 min. Each session of the telephone call was initiated by a review of the educational contents of the previous session and followed by the new contents. In addition to telephone training, educational materials were provided to the intervention group members in electronic files, and they were asked to apply the training items at home. To assess the effectiveness of telenursing on QoL of COVID-19 patients, a research colleague completed the QoL survey for both groups over the telephone after 1 month.

### Statistical analysis

The SPSS software version 16 was used for analysis. The Kolmogorov-Smirnov test was performed to investigate the distribution of quantitative variables. An independent *t* test and chi-square test were used to examine the homogeneity of the two groups. According to these tests, the intervention and control groups were not significantly different in demographic variables and were homogeneous. The paired *t* test and Wilcoxon test were used to compare within-group mean QoL scores of patients before and after the intervention. The independent *t* test and Mann-Whitney test were applied to compare the mean scores between the control and intervention groups in each study phase. The Pearson correlation test was employed to evaluate the correlation between age and QoL scores. Notably, the significance level was considered less than 0.05 in all tests.

## Results

The mean age of patients was 53.33 ± 9.87 years. The majority (62.5%) were males. Other demographic characteristics are summarized in Table 1.

**Table 1:** Demographic characteristics of patients in the intervention and control groups (Patients discharged from the 22nd Bahman Hospital of Khaf in 6 months since 2020)

Variable	Intervention group	Control group	Significance level
**Age**	Mean ± Standard deviation	Mean ± Standard deviation	***p*** **= 0.72**
	53.65 ± 10.11	53.01 ± 9.63	
**Gender**	Number (percent)	Number (percent)	***p*** **= 0.34**
**Woman**	23 (38.4)	22 (36.6)	
**Man**	37 (61.6)	38 (63.4)	
**Marital status**			***p*** **= 0.34**
**Single**	11 (18.3)	5 (8.3)	
**Married**	41 (68.3)	44 (73.3)	
**Divorced**	4 (6.6)	5 (8.3)	
**Spouse deceased**	4 (6.6)	6 (10)	
**Education**			***p*** **= 0.51**
**Primary**	33 (55)	35 (58.3)	
**Secondary**	4 (6.7)	8 (13.3)	
**High school**	18 (30)	13 (21.7)	
**Tertiary**	5 (8.3)	4 (6.7)	
**Occupation**			***p*** **= 0.22**
**Homemaker**	20 (33.3)	17 (28.3)	
**Employee**	7 (11.7)	2 (3.3)	
**Self-employed**	20 (33.3)	28 (46.7)	
**Retiree or unemployed**	13 (21.7)	13 (21.7)	

The mean scores of the eight QoL domains were not significantly different in intervention and control groups at baseline. However, 1 month after the educational intervention, the results showed a statistically significant difference between the two groups in terms of general health perceptions, social functioning, mental health, bodily pain, vitality, role limitations due to physical problems, and role limitations due to emotional problems (*p* <0.001), indicating a significant improvement in the mean score of QoL in the intervention group (Table 2).

**Table 2:** Comparison of eight domains of patients’ quality of life before and 1 month after intervention in the intervention and control groups (Patients discharged from 22nd Bahman Hospital of Khaf in 6 months since 2020)

Variable	Group	Before intervention	One-month after intervention	Paired ***t*** test or Wilcoxon test
		Mean ± Standard deviation	Mean ± Standard deviation	
**General mental health**	**Intervention**	47.59 ± 10.91	54.57 ± 7.83	*p* <0.001^a^
	**Control**	48.28 ± 7.53	46.49 ± 4.53	*p* = 0.16
	**Independent** ***t*** **test**	*p* = 0.68	*p* <0.001	
**Social functioning**	**Intervention**	68.83 ± 17.47	76.11 ± 10.88	*p* = 0.03^a^
	**Control**	70.83 ± 13.44	68.77 ± 10.70	*p* = 0.36
	**Independent** ***t*** **test**	*p* = 0.48	*p* <0.001	
**General health perceptions**	**Intervention**	63.55 ± 8.99	70.23 ± 7.60	*p* <0.001^a^
	**Control**	64.44 ± 11.31	61.11 ± 7.92	*p* = 0.04^a^
	**Independent** ***t*** **test**	*p* = 0.63	*p* <0.001	
**Vitality**	**Intervention**	61.04 ± 12.29	67.02 ± 10.36	*p* <0.001^a^
	**Control**	59.93 ± 11.73	58.11 ± 8.49	*p* = 0.46
	**Independent** ***t*** **test**	*p* = 0.61	*p* <0.001	
**Physical functioning**	**Intervention**	61.44 ± 16.73	67.40 ± 6.69	*p* <0.001^a^
	**Control**	60.50 ± 14.68	58.89 ± 6.91	*p* = 0.31
	**Independent** ***t*** **test**	*p* = 0.74	*p* <0.001	
**Bodily pain**	**Intervention**	60.66 ± 23.20	68.57 ± 13.67	*p* = 0.34
	**Control**	61.50 ± 22.15	55.89 ± 17.45	*p* = 0.15
	**Mann-Whitney test**	*p* = 0.75	*p* <0.001	
**Role limitations due to physical problems**	**Intervention**	83.75 ± 15.48	80.45 ± 8.50	*p* = 0.43
	**Control**	83.12 ± 16.40	76.30 ± 10.70	*p* = 0.10
	**Mann-Whitney test**	*p* = 0.89	*p* = 0.06	
**Role limitations due to emotional problems**	**Intervention**	64.2 ± 18.12	80.95 ± 5.90	*p* <0.001^a^
	**Control**	69.44 ± 18.19	74.47 ± 10.33	*p* = 0.08
	**Mann-Whitney test**	*p* = 0.09	*p* <0.001	

*p* is the *p* value reported by the Wilcoxon test

^a^Significant

In the intervention group, the Chi-square test showed a statistically significant relationship between the demographic variables of gender and marital status and the QoL score (*p* = 0.01 and *p* = 0.03, respectively), so that education through telenursing had a greater effect on increasing QoL score in men (73.72 ± 2.98) and married people (74.58 ± 4.64). There was a statistically significant and positive correlation between age and QoL score (*p* <0.001, *r*: 0.52). That is, the QoL level decreased with age.

Comparison of the overall QoL score of patients before and after the intervention in the intervention and control groups shows that the QoL of COVID-19 patients has increased significantly after the educational intervention using the telenursing process (*p* <0.001) (Table 3).

**Table 3:** Comparison of patients’ quality of life scores before and after the intervention in intervention and control groups (Patients discharged from 22nd Bahman Hospital of Khaf in 6 months since 2020)

Quality of life score Group	Mean ± Standard deviation		Paired t-test results
	Before intervention	After intervention	
**Control**	**64.75 ± 9.45**	**63.62 ± 3.93**	***p*** **= 0.44**
**Intervention**	**63.88 ± 9.67**	**72.62 ± 3.51**	***p*** **<0.001**^a^
**Independent** ***t*** **test result**	***p*** **= 0.61**	***p*** **<0.001**	

^a^Significant

## Discussion

In this study, we investigated the impact of telenursing training on the QoL of COVID-19 patients. The feature distinguishing the present study from similar ones is that other telenursing-related studies have examined QoL in patients with chronic diseases. In contrast, we have considered telenursing education for an acute respiratory disease (COVID-19), which stands as a global crisis.

Our results showed that the mean QoL level increased significantly in COVID-19 patients of the intervention group than in the control group. This can be due to the continuous follow-up program that involved training and bilateral relationship between the patient and the researcher during the intervention. Thus, training and support are two fundamental elements that are needed during the recovery period. In addition, telephone follow-up can be conducive to early diagnosis of complications and can help inform patients of the presence of complications so that appropriate treatment can be provided.

The results of this study are consistent with the results of Behzadet al.’s study who examined the effect of back teaching on the QoL of patients with myocardial infarction. They showed that performing three 45-min training sessions of face-to-face and telephone follow-up within a month can improve patients’ QoL [25]. Furthermore, irrespective of its time and place, telephone follow-up establishes a dynamic, flexible, and continuous care relationship between the nurse and the patient, improving patient QoL [26].

The present study results showed that at baseline, the mean scores of the eight QoL domains were not significantly different in the two groups. However, 1 month after the implementation of the educational intervention, the intervention group scored significantly higher in the domains of general health, social functioning, general mental health, bodily pain, vitality, physical function, and role limitations due to emotional problems than the control group (*p* <0.001). In addition, the results of Ebrahimi et al. study examining the effect of a family-centered intervention on QoL of patients with acute myocardial infarction showed that the mean scores of mental health, physical health, social health, and total QoL scores were significantly higher in the intervention group than the control group after the intervention [21].

The social aspects of the life of a COVID-19 patient were also addressed in the present study, and the training delivered through telenursing increased the QoL social dimension score in the intervention group compared to the control group. Lavazzo et al. also showed that telenursing in heart failure patients increases their QoL. In their study, patients’ QoL in terms of physical and social functioning increased significantly in the intervention group compared to the control group, which is in line with the results of our study [27].

In their study, Shojaei et al. examined the effect of telenursing on the level of hope in patients with heart failure, which corresponds with the psychological dimension of the present study. They stated that the psychological dimension of QoL can change under different conditions such as the type of disease, sense of weakness and helplessness, fear of death, and decreased performance, among others. Their results show that post-discharge training and follow-up can raise hope in heart failure patients [28]. Thus, the current study and Shojaei et al. are consistent be theirs the interval them covered and improved a psychological facet in patients.

Our findings showed a statistically significant relationship between QoL and the age of COVID-19 patients, such that QoL levels decrease with age. This makes sense given the many physical and psychological problems that older people face, which is compatible with Abbasi et al. study [29].

Also, in this study, there was a statistically significant relationship between patients’ gender and their QoL levels, such that men had higher QoL levels. In general, psychological factors, women’s different perceptions of disease symptoms, depression, and the level of social support can lead to this gender-based difference [30]. Additionally, women have lower vitality and physical function than men. This difference can become more pronounced after 1 year of follow-up because women are more likely than men to suffer from sleep disorders, emotional problems, decreased energy and power, and illness [31].

There was a statistically significant relationship between marital status and QoL in our participants, where married people reported higher QoL than single ones. To justify this, it can be said that the presence of the spouse who enhances the ‘patient’s sense of solidarity and belonging to others can increase the level of QoL in the social dimension.

### Limitations

Among the limitations of this study are relatively small sample size, sampling method, COVID-19-related psychological problems of patients, and individual and personality differences of patients at the time of data collection, which may have affected the study results. To control these issues, the samples were randomly assigned into intervention and control groups. However, it should be noted that it was out of the researcher’s capacity to control all constraints. It is recommended to conduct further qualitative and quantitative studies in different research environments using larger sample sizes for further studies.

## Conclusions

The results of this study have highlighted the importance of nurse-led telephone-based educational follow-up (telenursing) with a focus on the improvement of patients’ QoL. Therefore, it is suggested that healthcare managers adopt appropriate policies for the implementation of telenursing, which can reduce the number of repetitive referrals to the hospital and the health system, decrease the waste of time and costs imposed on the system, and improve QoL in COVID-19 patients.

## Data Availability

Data are available from the corresponding author on reasonable request.

## Abbreviations

WHO: World Health Organization
QoL: Quality of life
SF: Short-form
